# From Perception to Adaptation: A Comparative Study of Plant Regulatory Networks in Response to Heat and Waterlogging Stress

**DOI:** 10.3390/plants15020328

**Published:** 2026-01-21

**Authors:** Javed Iqbal, Sikandar Amanullah, Chengyue Li, Xiaohui Qin, Pengbo Yu, Xuanyang Chen, Dongliang Qiu

**Affiliations:** 1 College of Horticulture, Fujian Agriculture and Forestry University, Fuzhou 350002, China; iqbal468@aup.edu.pk (J.I.); 22303102001@fafu.edu.cn (C.L.); 2 Mountain Horticultural Crops Research and Extension Center, Department of Horticultural Science, North Carolina State University, Mills River, NC 28759, USA; sikandaraman@yahoo.com; 3 College of Agronomy, Fujian Agriculture and Forestry University, Fuzhou 350002, China; 13774898835@163.com (X.Q.); 15908889807@163.com (P.Y.); cxy@fafu.edu.cn (X.C.)

**Keywords:** heat stress, waterlogging stress, stress adaptation, ethylene, climate, transcriptomic, metabolomics, abscisic acid

## Abstract

Heat and waterlogging are critical abiotic stresses that threaten crop productivity, especially as climate change intensifies their frequency and severity. While both stresses independently disrupt essential physiological functions such as photosynthesis, respiration, and nutrient uptake, their underlying mechanisms and adaptive strategies exhibit key differences. This review presents a systematic comparison of plant responses to heat and waterlogging stress, focusing on both their shared and distinct impacts on plant physiology, biochemistry, and molecular regulation. We synthesize recent insights from omics technologies, including transcriptomic and metabolomics, to explore regulatory pathways, hormonal crosstalk (e.g., ABA–ethylene interactions), and metabolic shifts (e.g., fermentation vs. chaperone induction) that drive stress tolerance. This comparative analysis similarly demonstrates that effective plant resilience to climate extremes depends on the coordinated optimization of shared stress management hubs, such as antioxidant defense systems and hormonal crosstalk, together with the deployment of stress-specific adaptive strategies, including molecular chaperone induction under heat stress and anaerobic metabolic reprogramming under waterlogging. By integrating convergent and divergent regulatory pathways, this framework provides a mechanistic and conceptual guide for breeding and engineering crops with durable tolerance to multiple, increasingly co-occurring abiotic stresses.

## 1. Introduction

Climate change is reshaping the global stress landscape faced by crop plants, not only by intensifying individual abiotic stresses but also by increasing the frequency with which multiple extremes co-occur [1,2]. Among these, heat stress and waterlogging stress are emerging as a particularly disruptive combination, often occurring sequentially or simultaneously as a consequence of erratic precipitation patterns, extreme rainfall events, and rising temperatures [2,3]. Such compound stress scenarios pose a unique biological challenge, as plants are required to cope with fundamentally different primary stressors within overlapping temporal windows.

Heat stress primarily disrupts cellular homeostasis through protein denaturation, membrane instability, and impaired photosynthetic machinery, whereas waterlogging imposes oxygen deprivation, constraining aerobic respiration and forcing a metabolic shift toward anaerobic energy production [4]. Despite these distinct primary insults, both stresses converge on a common set of secondary dysfunctions, including oxidative stress, energy deficits, hormonal imbalance, and growth inhibition [3,5]. How plants integrate responses to these contrasting stress cues—while managing shared downstream consequences—remains a central but insufficiently synthesized question in plant stress biology. Most existing studies have examined heat and waterlogging stress in isolation, emphasizing stress-specific traits or tolerance mechanisms [4,5]. Over evolutionary time, plants have developed numerous structural, physiological, and biochemical mechanisms to withstand environmental challenges. Thanks to ex-tensive research efforts, significant progress has been made in identifying how plants perceive and respond to environmental stresses [6]. However, this siloed approach limits our ability to identify shared regulatory hubs and points of divergence that could be strategically exploited to enhance resilience to multiple stresses. A comparative perspective is therefore essential to understand whether plants rely on conserved stress management systems, such as antioxidant defenses and hormonal crosstalk, or whether effective tolerance requires the deployment of distinct molecular solutions, including heat shock protein-mediated proteostasis under heat stress and fermentation-driven energy maintenance under waterlogging [3,4].

**Figure 1:**
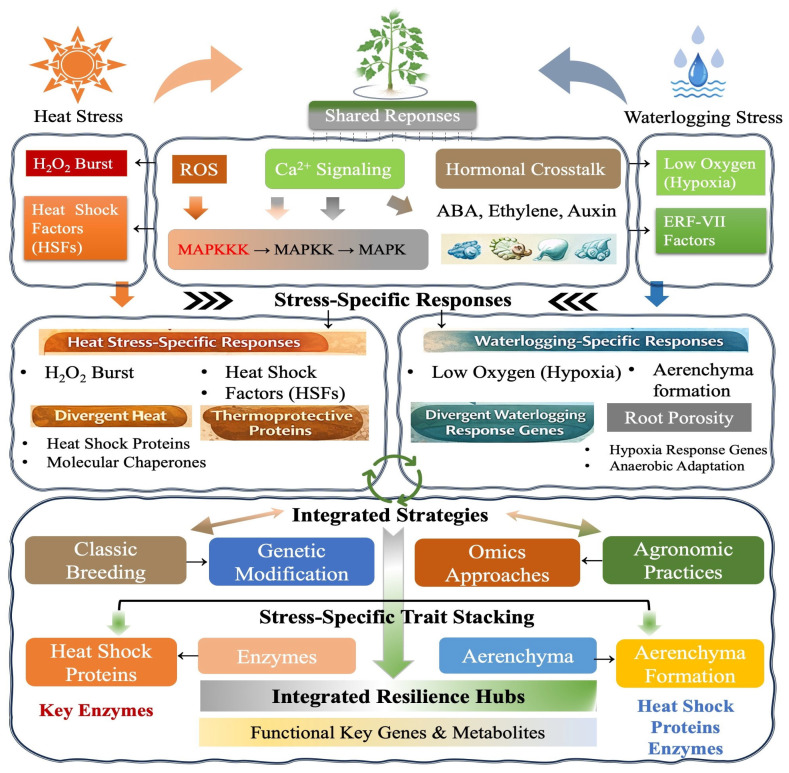
Comparative schematic overview of physiological and biochemical responses involved in both heat and waterlogging stress. It similarly emphasizes integrated strategies that combine genetic engineering, breeding, and cultural practices to improve plant resilience.

In this review, we tried to address this knowledge gap by presenting a systematic comparative analysis of plant responses to heat and waterlogging stress, spanning physiological, biochemical, and molecular regulatory layers [1,5]. By explicitly contrasting convergent and divergent adaptive strategies, we aim to identify key advantage points for crop improvement, providing a conceptual framework to guide breeding, genetic engineering, and agronomic interventions for developing crops resilient to increasingly complex climate extremes. The visual schematic presented below (Figure 1) provides a true conceptual model of the entire review, encapsulating the systems-level perspective. It clearly illustrates the overall plant responses towards both heat and waterlogging stresses and similarly highlights shared mechanisms, such as oxidative stress, ROS production and complex hormonal crosstalk, alongside stress-specific adaptations, including heat shock proteins (HSPs) under heat stress and aerenchyma formation under waterlogging stress. Additionally, it underscores integrated strategies that combine genetic engineering, breeding, and agronomic practices for enhancing plant resilience. The proposed schematic figure also acts as a foundational reference throughout the review, reinforcing the integrative narrative and providing clarity in understanding the complex responses to these environmental stresses.

## 2. A Comparative Framework: From Primary Stress Perception to Convergent Crises in Plants

It is essential to investigate the effects of major abiotic stresses (e.g., heat and waterlogging) that hinder plant growth and development due to their crucial physiological impact on plants. Heat stress in crop plants occurs when temperatures rise beyond a critical threshold for a duration long enough to cause irreversible damage to growth and physiological processes. Typically, a temperature increase of 10–15 °C above ambient levels is considered heat stress. However, the severity of heat stress depends on its intensity, duration, and the rate of temperature increase. The occurrence of heat stress in specific climatic zones is influenced by the frequency and duration of high temperatures during both day and night [7].

On the other hand, soil flooding imposes a multifaceted stress on plants, commonly referred to as either submergence or waterlogging, depending on the depth of the water table [8]. Flooding can be classified as waterlogging when the water level is shallow and only covers the root zone, or as submergence when water completely covers the aerial parts of the plant [9]. Waterlogging stress happens when the soil becomes excessively saturated with water, leading to anaerobic conditions in the root zone [10]. The frequency of waterlogging events is rising due to climate change, leading to significant crop loss. Globally, 10–12% of agricultural land is affected annually, with economic losses exceeding USD 74 billion [11,12]. Both heat and waterlogging stress are projected to increase in frequency and intensity, posing a critical challenge for crop production and food security in the face of global climate change.

Given the growing challenges posed by these stresses, it is crucial to understand their shared and distinct impacts on plant physiology and adaptive mechanisms towards resistance or susceptibility. In this section, we provide a comparative analysis of the effects of heat stress and waterlogging stress, focusing on the shared physiological and biochemical mechanisms and stress-specific adaptations. The discussion is organized around key processes such as photosynthesis, respiration, reactive oxygen species (ROS) production [13], and nutrient relations. These processes are integral to understanding the impact of both stresses on plants and are crucial for managing these environmental challenges.

**Figure 2:**
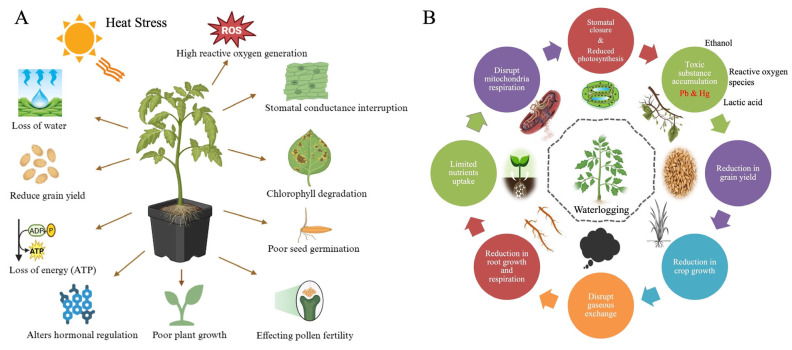
A general overview of comparative effects of (**A**) heat stress and (**B**) waterlogging stress on plants, respectively.

The figure provides a visual summary of the stress-specific effects of heat and waterlogging conditions on plants, separately (Figure 2). It illustrates the physiological and biochemical disruptions caused by both stresses, highlighting key impacts such as changes in photosynthesis, respiration, nutrient uptake, and energy metabolism, like stomatal closure and reduced plant growth, while distinguishing the main stress-specific effects, such as the accumulation of toxic metabolites under waterlogging and the generation of reactive oxygen species (ROS) under heat stress.

### 2.1. Primary Stress Perception and Early Cellular Disruption

Heat and waterlogging impose fundamentally distinct primary stresses, yet both rapidly destabilize cellular homeostasis. Under heat stress, elevated temperatures directly impair protein conformation, leading to widespread misfolding, aggregation, and loss of enzymatic activity [14,15]. In contrast, waterlogging primarily disrupts oxygen availability, suppressing mitochondrial respiration and triggering hypoxia-induced metabolic reprogramming [16,17]. Despite these differences, both stresses rapidly compromise membrane integrity, ion balance, and redox homeostasis, setting the stage for downstream systemic dysfunction [18]. This contrast underscores a central paradox in plant stress biology: distinct primary insults can converge on shared cellular crises, necessitating both common and specialized adaptive responses [19].

### 2.2. Energy Metabolism Under Thermal and Hypoxic Constraints

Energy metabolism represents one of the clearest points of divergence and convergence between heat and waterlogging stress. Heat stress elevates respiratory demand while simultaneously destabilizing mitochondrial enzymes, often resulting in inefficient ATP production and accelerated carbon depletion [15,20]. Waterlogging, by contrast, directly restricts oxidative phosphorylation due to oxygen scarcity, forcing reliance on glycolysis and fermentative pathways to sustain minimal energy supply [16,21]. While both stresses culminate in cellular energy deficits, the underlying constraints differ fundamentally, implying that strategies aimed at enhancing mitochondrial efficiency may preferentially improve heat tolerance, whereas strengthening fermentative capacity and carbon-use efficiency is more critical under waterlogging [17,22]. Recognizing these distinctions is essential for avoiding maladaptive trade-offs in multi-stress crop engineering [19].

### 2.3. Photosynthetic Dysfunction and Carbon Assimilation Failure

Photosynthesis is a major target of disruption under both stresses, though through different proximal mechanisms. Heat stress impairs photosystem II stability, Rubisco activase function, and thylakoid membrane organization, leading to rapid declines in carbon assimilation [23]. Waterlogging indirectly constrains photosynthesis through stomatal closure, carbohydrate feedback inhibition, and reduced sink demand under hypoxic root conditions [11]. Despite these mechanistic differences, both stresses result in diminished photosynthetic efficiency and altered source–sink relationships, reinforcing carbon limitation as a shared downstream constraint on growth and yield [24].

### 2.4. Oxidative Stress as a Convergent Cellular Crisis

Oxidative stress represents a shared downstream consequence of both heat and waterlogging stress, albeit arising through distinct mechanisms. Heat stress accelerates reactive oxygen species (ROS) production through impaired electron transport in chloroplasts and mitochondria [1,10], whereas waterlogging-induced hypoxia promotes ROS accumulation particularly during re-oxygenation phases [25]. Despite these differences, both stresses necessitate the rapid activation of enzymatic and non-enzymatic antioxidant systems to prevent irreversible cellular damage [25]. This convergence identifies redox regulation as a shared stress management hub and a strategically valuable target for enhancing broad-spectrum stress resilience [26].

### 2.5. Hormonal Reprogramming and Growth Trade-Offs

Hormonal signaling networks play a central role in coordinating growth-defense trade-offs under both stresses. Heat stress is strongly associated with altered abscisic acid (ABA), ethylene, and auxin signaling, reshaping thermo-tolerance and developmental plasticity [15,20]. Waterlogging similarly induces ethylene accumulation and modulates ABA and gibberellin balance to regulate adventitious root formation, shoot elongation, and survival under hypoxia [17,21]. While the hormonal players often overlap, their functional outcomes differ, underscoring the importance of context-dependent hormonal crosstalk in stress adaptation [24,26].

### 2.6. Strategic Implications: Shared Hubs vs. Stress-Specific Solutions

Collectively, this comparative analysis demonstrates that effective tolerance to compound heat and waterlogging stress cannot rely solely on reinforcing generalized stress responses. Instead, resilience emerges from the integration of shared regulatory hubs—such as antioxidant defenses, hormonal coordination, and redox signaling [19,22,26,27], with stress-specific molecular solutions, including proteostasis networks under heat stress and anaerobic metabolic capacity under waterlogging. This framework provides a mechanistic basis for prioritizing intervention points in breeding, genetic engineering, and agronomic strategies aimed at developing crops resilient to increasingly complex climate stress combinations.

The summary table (Table 1) provides a comparative overview of key physiological and biochemical processes affected by both heat and waterlogging stress. It presents a clear point-by-point comparison of these stresses across shared mechanisms such as oxidative stress, respiration, photosynthesis, and energy metabolism.

## 3. Plants Adaptations to Heat and Waterlogging Stress

Plants are frequently exposed to various abiotic stresses, including heat, cold, waterlogging, drought, and salinity, all of which can significantly impair their growth, development, and productivity. Among these, heat and waterlogging stresses are particularly detrimental, especially with the worsening effects of climate change, which has increased the frequency and intensity of such stresses [28]. While both stresses affect plant health, their mechanisms of action and adaptation are distinct, requiring separate considerations. To survive and maintain functionality under such adverse conditions, plants have evolved a complex defense system comprising physiological, biochemical, and molecular mechanisms [29,30].

Herein, we explored the individual adaptations to heat and waterlogging stress separately, highlighting both the contrasts and shared mechanisms involved in each stress response. These adaptations not only enable plants to tolerate stress but also help minimize damage at the cellular and systemic levels. Understanding these distinct and overlapping strategies will provide insights into how plants cope with these environmental challenges, paving the way for effective management strategies in agriculture. In the following sections, we will discuss these mechanisms individually to better understand how plants respond to heat and waterlogging stress.

### 3.1. Physiological Mechanisms of Plants in Responses to Heat and Waterlogging Stress

Both heat stress and waterlogging stress disrupt key physiological processes such as water transport, gas exchange, and overall growth, often leading to symptoms like leaf wilting, yellowing, and reduced growth [31]. In response, plants adjust key physiological processes, including stomatal regulation to control water loss [32], modifications in root structures to enhance water and oxygen uptake [33], and the formation of air-filled tissues, such as aerenchyma, to survive in flooded soils [34]. These adaptations are critical in helping plants withstand short-term stress and provide a base for longer-term biochemical and genetic responses [14]. The following sections will explore each of these mechanisms in detail to understand their roles in stress tolerance.

#### 3.1.1. Stomatal Regulation

**Figure 3:**
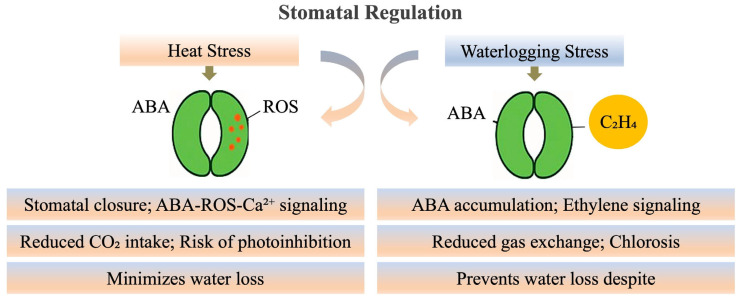
Schematic illustration of distinct hormonal and physiological pathways regulating stomatal behavior under heat and waterlogging stress.

Stomata are microscopic pores on the surface of leaves that play a critical role in controlling gas exchange and transpiration. Stomatal closure in response to both stresses serves as a rapid defense mechanism to preserve internal water balance and prevent desiccation (Figure 3). Although essential for short-term survival, prolonged stomatal closure compromises energy production and growth, as it limits photosynthetic activity [26].

A: *Stomatal Regulation under Heat Stress*

Under heat stress, plants typically respond by closing their stomata to conserve water, although this also restricts carbon dioxide intake, which can reduce photosynthesis [35]. In high-temperature environments, increased vapor pressure deficit (VPD) elevates water loss via transpiration, triggering stomatal closure as an immediate defense response [31,36]. This response is largely mediated by ABA, a stress-responsive hormone that activates SnRK2 protein kinases and ion channels such as SLAC1, leading to changes in guard cell turgor and stomatal pore closure [37]. In this process, reactive oxygen species (ROS), notably hydrogen peroxide (H_2_O_2_), serve as signaling molecules that modulate calcium ion (Ca^2+^) influx and strengthen the ABA signaling pathway in guard cells [13].

B: *Stomatal Regulation under Waterlogging Stress*

In waterlogging, stomatal closure is also observed but is primarily due to root hypoxia and reduced water uptake. The hypoxic conditions in waterlogged soils alter hormonal balance, typically increasing ABA and ethylene levels, both of which promote stomatal closure. This ABA–ethylene crosstalk is a key feature of the waterlogging response [38,39]. Additionally, waterlogging reduces cytokinin biosynthesis, enhancing stomatal sensitivity and contributing to premature leaf senescence [40]. The stomatal closure in waterlogging stress helps minimize water loss, but also restricts gas exchange and can limit photosynthesis.

C: *Shared Mechanisms and Comparative Analysis of Stomatal Regulation under Waterlogging and Heat Stress*

Both heat stress and waterlogging stress involve ABA (abscisic acid) as a central mediator of stomatal closure [41,42]. Under heat stress, ABA helps plants conserve water by closing the stomata, thereby reducing water loss through transpiration [43]. This mechanism is vital for water conservation during periods of high temperature and increased vapor pressure deficit (VPD). In contrast, under waterlogging stress, ABA regulates stomatal closure in response to hypoxia (low oxygen) in the roots [44], which reduces the plant’s ability to absorb water.

Although ABA signaling is a shared mechanism, the triggers and regulatory pathways for stomatal closure differ between the two stresses. In heat stress, ABA and ROS signaling play a prominent role in controlling stomatal closure to prevent water loss under high temperatures. In waterlogging stress, ABA is also involved in stomatal regulation, but ethylene and hypoxia signaling dominate, especially in root adaptations like aerenchyma formation and increased water uptake. A comparison of stomatal regulation under heat and waterlogging stress is presented in Table 2, which summarizes the primary triggers, key hormones involved, mechanisms of closure, and the adaptive benefits and long-term limitations of these responses. Understanding these dynamic signaling pathways is critical for improving crop resilience through targeted breeding and genetic strategies.

The schematic Figure 3 indicating the that heat stress triggers ABA–ROS–Ca^2+^ signaling that induces stomatal closure, reducing CO_2_ intake and risking photoinhibition but minimizing water loss. In contrast, waterlogging stress involves ABA accumulation and ethylene signaling, leading to impaired gas exchange and chlorosis while preventing excessive water loss.

#### 3.1.2. Structural Adaptations of Roots

**Figure 4:**
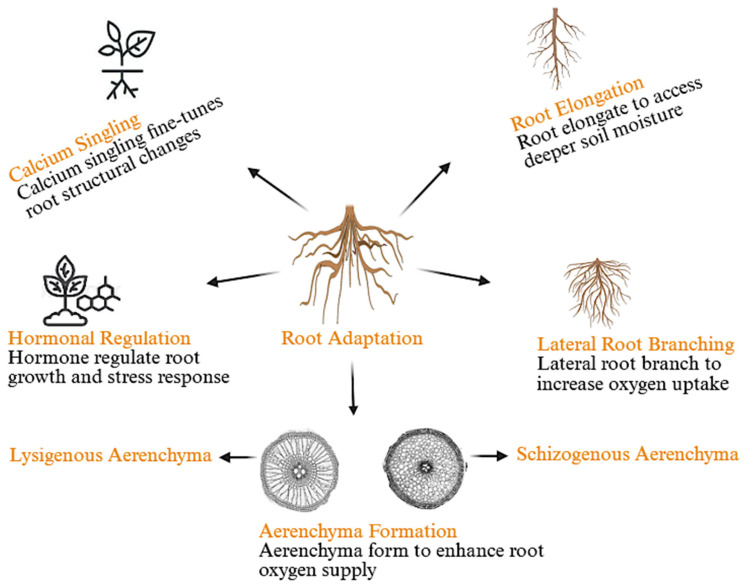
Root adaptations in response to waterlogging and heat stress. Key root mechanisms activated under these stress conditions include root elongation to access deeper soil moisture, lateral root branching for enhanced oxygen uptake, aerenchyma formation to improve internal oxygen supply, hormonal regulation of root growth and stress responses, and calcium signaling to fine-tune root structural changes.

In addition to stomatal regulation and aerenchyma formation, plants also modify their root systems to effectively respond to environmental stresses such as heat and waterlogging. Root architecture is essential for a plant’s ability to take up water, oxygen, and nutrients, especially when facing stress. Waterlogging creates a hypoxic environment in the root zone, which impairs aerobic respiration [45,46]. In contrast, heat stress increases water loss through evaporation and raises soil temperatures [47], both of which challenge the plant’s ability to maintain hydration. To cope with these stresses, plants adjust their root systems by elongating roots, increasing lateral root branching, and enhancing root porosity through the formation of aerenchyma (schizogenous and Lysigenous). These root adaptations are critical for maintaining water balance, optimizing gas exchange, and supporting overall growth under stress conditions. The root adaptations are illustrated in Figure 4, which highlights the key mechanisms activated under waterlogging and heat stress conditions, including root elongation, lateral root branching, and aerenchyma formation.

A: *Structural Adaptation of Roots under Heat Stress*

Under heat stress, plants elongate their roots to access deeper, cooler, and moister soil layers, which are crucial for water retention. This adaptation is driven by ABA (abscisic acid), which is produced in response to heat and regulates deeper root growth [48]. Additionally, calcium (Ca^2+^) signaling fine-tunes root structural changes, allowing plants to better adapt to the fluctuating water availability during high temperatures [49]. The root elongation under heat stress primarily serves to optimize water uptake in deeper soil layers.

B: *Structural Adaptation of Roots under Waterlogging Stress*

In waterlogging stress, roots are exposed to hypoxia, reducing their ability to absorb oxygen and nutrients [50]. To address this situation, plants elongate roots and increase lateral root branching, enhancing root surface area for oxygen uptake. The formation of aerenchyma within the roots improves oxygen diffusion, enabling plants to survive under waterlogged conditions [51]. This response is strongly regulated by ethylene, which promotes root elongation and aerenchyma formation under oxygen-limited environments [26]. Moreover, Calcium (Ca^2+^) plays a crucial role in the plant’s response to waterlogging stress by acting as a secondary messenger in the signaling pathways [52].

C: *Shared Mechanisms and Comparative Analysis of Root Adaptation under Heat and Waterlogging Stress*

Plants respond to heat stress and waterlogging stress by modifying their root systems to cope with changes in water availability and environmental conditions. Both stresses activate ABA (abscisic acid) signaling to regulate root growth and help maintain water balance, with calcium (Ca^2+^) signaling playing a key role in fine-tuning these adaptations. However, the regulatory pathways differ between the two stresses. Under heat stress, ABA promotes deeper root growth to access moisture [48].

While in waterlogging stress, ABA and ethylene together regulate root elongation and aerenchyma formation to improve oxygen uptake in hypoxic conditions [39]. Heat stress primarily triggers root elongation through ABA, supported by ROS (reactive oxygen species) signaling to help cope with oxidative damage [53]. In contrast, waterlogging stress induces lateral root branching and aerenchyma formation through ABA and ethylene signaling, which enables the plant to survive under low-oxygen conditions.

#### 3.1.3. Aerenchyma Formation Under Waterlogging Stress

Waterlogging stress severely restricts oxygen availability in the root zone, creating hypoxic or anoxic conditions that impair respiration and energy production in plant tissues. To overcome this challenge, many plant species form aerenchyma, specialized airy tissue in adventitious roots that facilitates gas transport between roots and shoots. Aerenchyma formation is a common adaptive trait associated with waterlogging tolerance [54]. It enhances gas exchange, reduces the metabolic energy required for root maintenance, and sustains plant growth under flooding conditions [55].

Aerenchyma are further classified into two types, schizogenous, which forms through the separation of adjacent cells, and lysigenous, which develops from the selective degradation or lysis of cortical cells (Table 3) [56]. Lysigenous aerenchyma is particularly prominent in the root cortex of several major cereal crops such as rice, maize, wheat, and barley. In wetland, for rice species, this type of aerenchyma is formed even under aerated soil conditions and its performance is further enhanced when exposed to waterlogged environments. In contrast, in upland cereals like maize, wheat, and barley, lysigenous aerenchyma formation is not constitutive but is specifically triggered by waterlogging stress [57]. The comparative research on aerenchyma formation across species has shown that rice and maize exhibit distinct responses to waterlogging stress. Rice (*Oryza sativa*), being a flood-tolerant species, forms aerenchyma constitutively in response to flooding, even under well-oxygenated conditions, which enhances its adaptation to anaerobic environments. In contrast, maize (*Zea mays*), a less flood-tolerant species, forms aerenchyma more slowly and primarily in response to waterlogging stress, suggesting less constitutive adaptation to anaerobic conditions [58,59]. The development of aerenchyma and increased root porosity are key adaptive traits that enhance plant tolerance to waterlogged condition [60]. For example, the proportion of aerenchyma formation in the total root cross-sectional area was much higher in rice roots compared to maize roots, indicating that rice, as a wetland species, has a greater capacity for oxygen transport in flooded environments [61].

The formation of lysigenous aerenchyma is regulated by the interplay between reactive oxygen species (ROS) and the gaseous hormone ethylene, which together initiate programmed cell death in specific cortical cells [62]. Additionally, nitric oxide (NO) and calcium (Ca^2+^) signaling pathways are involved in modulating aerenchyma development, working alongside ethylene to coordinate cell death and gas space expansion [63]. Ethylene accumulates in root tissues due to restricted gas diffusion and the stimulation of its biosynthesis under waterlogged conditions. Simultaneously, elevated ROS levels act as signaling molecules but can also cause cellular damage. To mitigate these effects, plants activate antioxidant defense systems as a protective response to oxidative stress induced by waterlogging [64].

### 3.2. Antioxidant Defense Systems Against Heat and Waterlogging Stress

**Figure 5:**
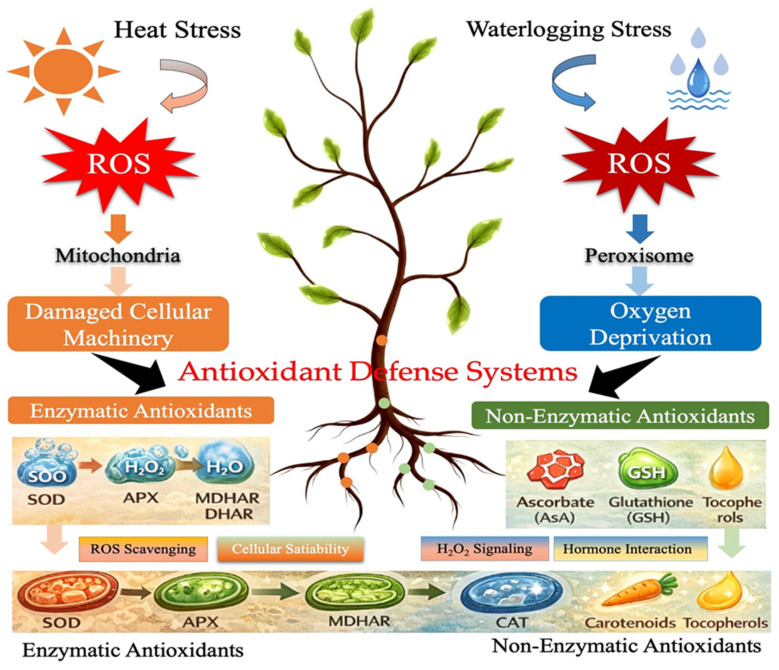
Comparative antioxidant defense systems against heat and waterlogging stress.

In light of the impacts of heat and waterlogging stress, it is crucial to explore how plants activate their antioxidant defense systems to combat the harmful accumulation of reactive oxygen species (ROS), which are produced during these stress conditions. Under heat stress, ROS primarily result from elevated temperatures that damage cellular machinery, leading to oxidative stress [65]. In contrast, under waterlogging stress, ROS are primarily generated due to oxygen deprivation, disrupting cellular functions, particularly in the roots, due to hypoxia [64]. The accumulation of ROS disrupts various physiological and metabolic processes, contributing to cellular damage and compromised plant function [13]. To counteract these effects, plants have evolved sophisticated enzymatic and non-enzymatic antioxidant mechanisms (Figure 5) that collectively reduce oxidative stress and help maintain cellular integrity. Both abiotic stresses induce the generation of reactive oxygen species (ROS), which are mitigated by enzymatic antioxidants (superoxide dismutase (SOD), ascorbate peroxidase (APX), catalase (CAT)) and non-enzymatic antioxidants (ascorbate, glutathione). These defense mechanisms help scavenge ROS, stabilize cellular structures, mediate hydrogen peroxide (H_2_O_2_) signaling, and facilitate hormone interactions, ultimately enhancing plant tolerance to stress

Enzymatic antioxidants play a critical role in neutralizing ROS by converting them into less harmful compounds. Key enzymes involved in this process include superoxide dismutase (SOD), ascorbate peroxidase (APX), catalase (CAT), monodehydroascorbate reductase (MDHAR), glutathione reductase (GR), dehydroascorbate reductase (DHAR), glutathione peroxidase (GPX), and various peroxidases (POX). These enzymes work in a coordinated manner within plant cells, where ROS generation and scavenging occur in specific organelles, ensuring cellular stability and function even under stress conditions [20,66]. In addition to enzymatic antioxidants, non-enzymatic antioxidants such as ascorbate (AsA), glutathione (GSH), carotenoids, and tocopherols play a pivotal role in ROS scavenging [21]. These small metabolites directly protect cellular components from oxidative damage, enhancing the plant’s ability to tolerate stress [67].

A central feature of the antioxidant defense system is the AsA-GSH pathway, which relies on NADPH as a crucial energy source for its operation. This pathway is vital for maintaining redox balance within the plant cell, particularly under stress. However, when NADPH levels are depleted, the capacity of these antioxidant pathways to mitigate ROS toxicity is compromised, further exacerbating oxidative stress [68]. Among the various ROS (Table 4), hydrogen peroxide (H_2_O_2_) has garnered significant attention due to its dual role as both a toxic agent and a signaling molecule. While, it is important to note that under heat and waterlogging stress, H_2_O_2_ plays a crucial role in modulating metabolic pathways and facilitating stress-induced gene expression under heat and waterlogging stress [69].

### 3.3. Comparative Mechanisms of Plant Responses to Heat and Waterlogging Stress

With the increasing frequency and severity of climatic extremes, plants face growing exposure to abiotic stress conditions, such as prolonged heat waves and episodic waterlogging. These stresses severely impact plant growth by disrupting cellular homeostasis, reducing photosynthetic efficiency, and causing damage to essential macromolecules, including proteins, lipids, and nucleic acids [72]. To adapt to these challenges, plants have evolved intricate molecular networks that sense, decode, and respond to these environmental cues [73].

These networks consist of highly coordinated processes that begin with the perception and transduction of stress signals, followed by precise transcriptional regulation, and are ultimately integrated through hormonal signaling pathways [74,75]. This complex regulatory system enables plants to mount an effective defense against stress, ensuring survival and maintaining plant health.

As mentioned in the introduction, Figure 1 serves as a dedicated conceptual figure that integrates signaling pathways, transcriptional control, and hormonal interactions for both heat and waterlogging stresses, clearly highlighting the points of convergence and divergence in these stress responses. Thus, the following sections will explore each of these mechanisms in detail to highlight their roles in conferring stress tolerance. Understanding these molecular level adaptations is essential for developing stress-resilient crops through advanced breeding and biotechnological approaches.

### 3.4. Adaptive Strategies of Plants Towards Stress Conditions

#### 3.4.1. Stress Perception and Signal Transduction

**Figure 6:**
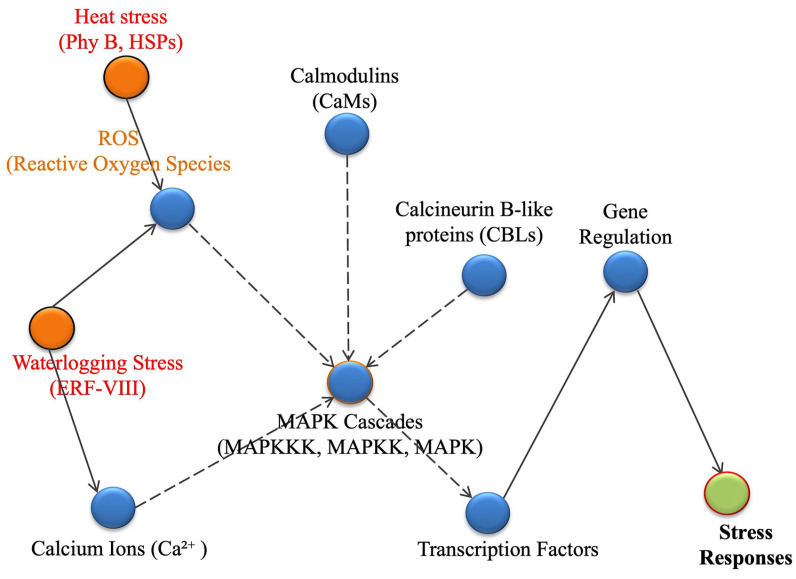
Signal perception and transduction pathways in response to heat and waterlogging stress. Orange circles are depicting the primary phase of heat and waterlogging stress; however, connected arrows with blue circles are exhibiting the associated pathways involved in final stress response behavior.

The plant’s ability to respond to abiotic stress begins with the precise perception of environmental signals, which is mediated through multiple mechanisms. The key components of signal transduction pathways such as heat shock proteins (HSPs), reactive oxygen species (ROS), calcium ions (Ca^2+^), MAPK cascades (MAPKKK, MAPKK, MAPK), and transcription factors coordinate in a proper way towards the plant’s response to these environmental stressors (Figure 6).

Under heat stress, plants detect thermal fluctuations through alterations in membrane fluidity and protein conformation. One key mechanism involves the temperature-dependent inactivation of photoreceptors, such as phytochrome B (phyB), which undergoes structural changes in response to elevated temperatures. These thermo-sensory signals converge on Heat Shock Transcription Factors (HSFs), which activate the expression of Heat Shock Proteins (HSPs). HSPs act primarily as molecular chaperones, assisting in refolding denatured proteins caused by heat stress and helping to maintain cellular integrity. HSPs are activated following the perception of heat stress, but they do not directly participate in sensing the stress signals or initiating regulatory feedback loops [76,77,78]. Understanding the molecular mechanisms of heat stress perception, such as the role of phytochrome B in detecting temperature fluctuations and the activation of HSFs, provides valuable insights for breeding heat-tolerant crops. By selecting varieties with enhanced HSP expression and improved protein refolding capabilities, breeders can improve heat resilience and maintain cellular integrity under high temperatures.

In contrast, waterlogging imposes oxygen deprivation (hypoxia), which is sensed through several mechanisms, including the stabilization of group VII Ethylene Response Factor (ERF-VII) transcription factors. Under normoxic conditions, ERF-VIIs are destabilized through the N-degron pathway, a proteolytic degradation mechanism. However, during hypoxia, the stabilization of ERF-VIIs activates genes associated with anaerobic metabolism, such as those involved in fermentation and glycolysis [79,80,81]. The PDC-ADH pathway plays a central role in this adaptive response, where pyruvate decarboxylase (PDC) catalyzes the conversion of pyruvate into acetaldehyde, and alcohol dehydrogenase (ADH) then reduces acetaldehyde to ethanol. This pathway enables plants to generate ATP under low-oxygen conditions, though less efficiently than aerobic respiration [82]. ERF-VII transcription factors regulate this pathway by activating the expression of PDC and ADH, allowing the plant to maintain energy production in hypoxic environments [83]. Additional hypoxia perception mechanisms include shifts in the cellular redox state, nitric oxide (NO) signaling, and mitochondrial dysfunction, which lead to the accumulation of signaling intermediates like ROS and NADPH [84]. Understanding the role of ERF-VII transcription factors in hypoxia perception and PDC-ADH pathway activation under waterlogging stress offers valuable insights for genetic engineering. By manipulating ERF-VII activity and enhancing the anaerobic metabolism pathway, crops could be developed to improve energy production and survival in waterlogged environments, ultimately improving waterlogging tolerance.

Once stress is perceived, the signal is transduced through conserved biochemical messengers to activate downstream molecular responses. Key signaling components include calcium ions (Ca^2+^), reactive oxygen species (ROS), and mitogen-activated protein kinase (MAPK) cascades (Figure 6). Calcium serves as a universal second messenger, with stress-induced fluctuations in cytosolic Ca^2+^ beings decoded by calcium-binding proteins such as calmodulins (CaMs) and calcineurin B-like proteins (CBLs), which in turn regulate specific kinases and gene expression [85]. Simultaneously, ROS—although potentially damaging at high concentrations—serve as critical signaling molecules that modulate transcription factor activity and activate antioxidant defense mechanisms. Generated primarily in chloroplasts, mitochondria, and by NADPH oxidases (RBOHs), which are enzymes, ROS act as both destructive agents and essential messengers in stress signaling. While high concentrations of ROS can damage cellular components, localized ROS waves generated by RBOHs serve as signaling molecules that activate stress-related transcription factors and regulate antioxidant gene expression. This controlled production of ROS is crucial for stress adaptation at the molecular level, helping plants coordinate their responses to environmental stress [86,87].

H_2_O_2_, in particular, plays a key role in ROS signaling under heat and waterlogging stress. It interacts with plant hormones, such as ethylene (ET) and abscisic acid (ABA), to regulate stress responses. These interactions enhance stress tolerance by modulating gene expression and cellular responses, helping the plant acclimate to adverse environmental conditions [71]. Furthermore, H_2_O_2_ influences calcium (Ca^2+^) and MAPK pathways, contributing to downstream signaling cascades that modulate stress responses. These interactions help activate necessary stress-related pathways, promoting tolerance and survival [70]. The involvement of endogenous H_2_O_2_ in enhancing tolerance to abiotic stress has prompted increasing interest in the exogenous application of H_2_O_2_ as a potential strategy for improving plant resilience [88].

MAPK cascades further amplify the stress signal by phosphorylating target proteins involved in gene regulation, cellular repair, and programmed cell death [89,90]. Mitogen-activated protein kinase (MAPK) cascades play a pivotal role in how plants respond to stress, acting as a central hub for signal transduction. These cascades—comprising MAP kinase kinase kinases (MAPKKKs), MAP kinase kinases (MAPKKs), and MAP kinases (MAPKs), that activate one another in a chain reaction through phosphorylation, leading to the activation of transcription factors that govern genes involved in defense, growth, and stress adaptation [91]. Specific MAPK pathways, like the MPK3/6 module, have been shown to be crucial in plant responses to heat and hypoxia. These pathways are also involved in the cross-talk between different hormones, helping coordinate the plant’s response to multiple stress signals simultaneously [92]. Together, these integrated signaling pathways allow for a rapid, specific, and coordinated response to environmental stress, enabling plants to prepare for the transcriptional and physiological changes necessary for survival.

#### 3.4.2. Transcriptional Reprogramming and Stress-Responsive Genes

**Figure 7:**
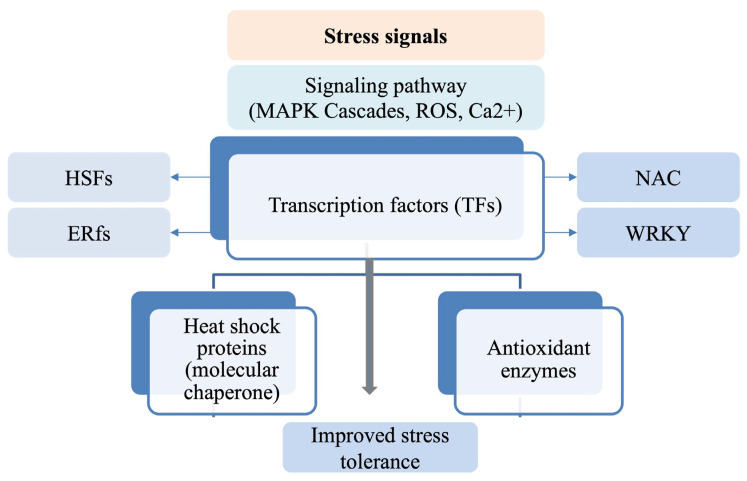
Transcriptional regulation and stress-responsive gene activation in plants under heat and waterlogging stress.

Once stress signals are perceived and transduced within the plant cell, the next essential step in plant stress responses is the activation of stress-responsive genes through transcriptional regulation (Figure 7). Transcription factors (TFs) are the key players in this process. These specialized proteins bind to specific DNA sequences, typically in the promoter or enhancer regions of target genes, acting as switches that either activate or repress genes critical for stress tolerance [93]. These TFs are typically triggered by earlier signaling events mediated by pathways such as MAPK cascades, ROS, and Ca^2+^, enabling precise gene expression in response to stress signals [94].

Two key families of transcription factors activated under heat and waterlogging stress are Heat Shock Factors (HSFs) and Ethylene Response Factors (ERFs). In heat stress, HSFs play a pivotal role in regulating the expression of Heat Shock Proteins (HSPs), which function as molecular chaperones to refold denatured proteins caused by elevated temperatures. This process helps maintain protein stability and prevents cellular damage under heat stress [95]. While the general role of HSFs in the heat stress response is conserved across species, the regulatory mechanisms can vary significantly. For example, in Arabidopsis, HSF activation is primarily regulated through the mitogen-activated protein kinase (MAPK) pathway, which modulates the expression of HSPs as part of the stress signal transduction process [96]. In contrast, in rice, HSF regulation is influenced by abscisic acid (ABA) signaling, which plays a critical role in coordinating the plant’s response to heat stress [97]. This highlights a distinct aspect of HSF signaling in rice, illustrating the species-specific nature of HSF regulation.

In contrast, ERFs (ERF-VII and ERF-VIII TFs) are critical under hypoxic conditions, such as those induced by waterlogging stress. They regulate responsive-genes and enzymes involved in anaerobic metabolism and aerenchyma formation, including alcohol dehydrogenase (ADH) and pyruvate decarboxylase (PDC), which are essential for plant survival in low-oxygen environments [98,99]. In addition to HSFs and ERFs, other transcription factor families, such as NAC and WRKY also play pivotal roles in stress regulation. NAC transcription factors are involved in a wide range of stress responses, including oxidative stress, programmed cell death, and root growth during waterlogging [100]. The WRKY family, known for its involvement in both biotic and abiotic stress responses, regulates the expression of genes related to stress tolerance, antioxidant activity, and cellular defense [101]. These transcription factors help activate a suite of stress-responsive genes that are involved in various protective mechanisms. For example, genes encoding antioxidant enzymes such as superoxide dismutase (SOD), catalase (CAT), and peroxidase (POD) are upregulated to mitigate ROS-induced damage [102]. Furthermore, plants activate osmotic adjustment mechanisms through the expression of genes involved in the synthesis of compatible solutes, such as proline and sugars, which help maintain cellular turgor under stress [103]. In response to waterlogging, cell wall remodeling genes are activated, such as those encoding pectin methyl-esterase and expansions, to allow the plant to adapt to swelling and changes in cell volume due to excess water [104].

This transcriptional reprogramming is essential for the plant’s ability to withstand and recover from abiotic stress. It enables plants to adjust their metabolic processes, protect cellular integrity, and sustain growth even under challenging conditions. Moreover, the regulation of stress-responsive genes equips plants not only to survive immediate stress but also to build resilience against future stress events, underscoring the importance of gene networks in shaping plant stress tolerance.

Mostly, the shared crosstalk occurs through ROS-sensitive TFs and ABA-responsive elements, integrating both heat and waterlogging responses. This allows for coordinated activation of antioxidant enzymes and stress-protective metabolites.

#### 3.4.3. Hormonal Crosstalk and Regulatory Networks

Hormonal crosstalk and regulatory networks are integral to the molecular mechanisms through which plants respond to abiotic stresses, such as heat and waterlogging. Phytohormones, including abscisic acid (ABA), ethylene, gibberellins (GA), auxins, and cytokinin, regulate a range of physiological processes that enable plants to adapt to adverse environmental conditions (Table 5). These hormones frequently interact in complex crosstalk networks, where their effects can either support or counteract one another, depending on the type of stress and the plant’s developmental stage [105]. The proper balance and coordination of these signals are essential for optimizing plant adaptation, ensuring survival under stress while maintaining overall growth [106].

ABA is a key hormone activated in response to both heat and water stress. Under heat or drought conditions, ABA promotes stomatal closure, reducing water loss through transpiration and aiding in moisture conservation. It also regulates root growth and osmotic adjustments, which are crucial for maintaining cell turgor during water scarcity [48,107]. In contrast, ethylene plays a central role during hypoxic conditions, such as waterlogging. It regulates the formation of aerenchyma tissue, improving oxygen exchange in the roots and enabling survival in low-oxygen environments [108]. While ABA and ethylene often work synergistically, their interactions can be complex; ABA primarily promotes stress tolerance, while ethylene facilitates specific adaptations to low-oxygen conditions [109].

Other hormones, including gibberellins (GA), auxins, and cytokinin, also contribute to stress adaptation. Gibberellins are typically associated with promoting growth, but their activity is reduced during stress to conserve resources and limit excessive growth [110]. Auxins regulate root architecture, promoting root elongation and lateral root development under waterlogged conditions, which enhances water and nutrient uptake [111]. Cytokinin, which influence cell division and shoot growth, support stress mitigation by promoting cellular repair and activating stress tolerance genes [112].

Additionally, hormonal signaling plays a significant role in stress memory, allowing plants to prepare for future stress events. The interactions between different hormones activate priming mechanisms, making the plant more responsive to future stresses. This priming mechanism is particularly beneficial in unpredictable environments where multiple stresses may occur in succession [113,114]. Moreover, feedback loops within hormonal networks enable precise regulation, where changes in gene expression can modulate the production and activity of specific hormones, helping the plant to fine-tune its response to stress.

However, in the shared hormonal crosstalk, ROS and calcium serve as central mediators for integrating multiple hormonal signals, allowing plants to fine-tune growth, metabolism, and stress tolerance simultaneously under both stress conditions.

#### 3.4.4. Physiology Responses and Morphological Outcomes

Finally, the above mentioned molecular and hormonal signals translate into protective physiological adjustments responses in plants. In heat stress, responses include enhanced membrane stability, protein stabilization via HSPs, and stomatal regulation to reduce transpirational water loss. In waterlogging stress, adaptations include development of aerenchyma for oxygen diffusion, adventitious root formation, and activation of anaerobic metabolism. However, both stresses include shared antioxidant defense systems, osmoprotectant accumulation, and upregulation of ROS-scavenging enzymes to mitigate oxidative damage.

Regarding the morphological outcomes, stress perception and signaling culminate in structural changes that enhance plant survival. In heat stress, leaf curling, altered root-to-shoot ratios, and heat acclimation traits improve thermos-tolerance. In waterlogging stress, stem elongation, hypertrophied lenticels, and increased root porosity facilitate oxygen transport. However, the shared responses include structural adaptations such as strengthened cell walls, improved vascular integrity, and root system adjustments support tolerance under both stresses.

The entire comparative framework highlights both stress-specific and shared adaptive mechanisms. By tracing the sequence from perception through transcriptional reprogramming, hormonal crosstalk, physiological responses, and morphological outcomes, it clarifies the hierarchical organization of plant stress responses and allows direct comparison of critical regulatory hubs, including HSFs versus ERF-VII TFs and calcium/ROS/MAPK signaling pathways.

## 4. Strategies for Enhancing Plant Tolerance to Heat and Waterlogging Stress

The ability of plants to withstand environmental stresses, particularly heat and waterlogging, is a key determinant of crop performance under changing climatic conditions. As highlighted in the previous sections, plants utilize a complex array of physiological, biochemical, and molecular mechanisms to cope with these stresses. However, these natural mechanisms can often be insufficient under extreme conditions. To bridge this gap, targeted strategies are required to strengthen plant resilience.

In this section, we summarize major approaches currently employed to enhance tolerance, specifically through breeding, genetic engineering, transcriptomic and metabolomic tools, and agronomic practices. Breeding and genetic engineering aim to create stress-resilient genotypes, while transcriptomic and metabolomic tools provide system-level insights for identifying key regulatory networks and metabolic pathways. Agronomic practices complement these efforts by offering field-based solutions that improve plant performance under adverse conditions. By building on our understanding of how plants respond to heat and waterlogging, these strategies offer exciting possibilities for developing crops that can thrive and continue to produce even in the face of tough environmental conditions.

**Figure 8:**
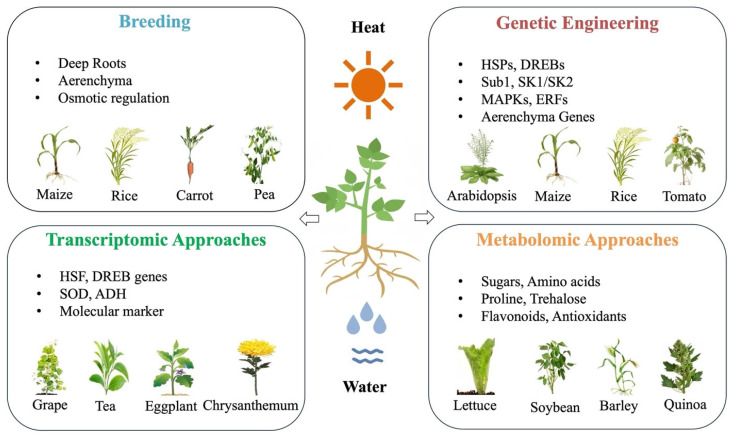
Integrated approaches to enhance plant tolerance to heat and waterlogging stress. This figure provides an overview of four key strategies—breeding, genetic engineering, transcriptomic, and metabolomic approaches—that contribute to improving plant resilience under heat and waterlogging stress.

As illustrated in Figure 8, these strategies are most effective when integrated, highlighting the importance of combining genetic, molecular, and agronomic interventions. Such a multidisciplinary framework provides a pathway toward developing crops with improved adaptability and sustained output under the increasing frequency of heat and waterlogging events driven by climate change.

### 4.1. Breeding for Stress Tolerance

Traditional breeding remains one of the most reliable and effective approaches for improving stress tolerance in crops. It focuses on selecting plants that naturally exhibit desirable traits, which are then passed down to subsequent generations. In the case of heat and waterlogging stress, breeders focus on selecting for traits such as root architecture, osmotic regulation, and hormonal signaling pathways. These traits help plants adapt to heat and waterlogging stress by optimizing their water and nutrient use, regulating their internal osmotic balance, and controlling physiological processes like transpiration [115].

Root traits are particularly critical. Plants with deep root systems can access water stored deep in the soil during drought conditions, while roots with aerenchyma allow for better oxygen uptake in waterlogged soils, improving root function and survival [55]. In addition to trait selection, breeders also take advantage of hybrid vigor (heterosis), where hybrid plants—resulting from the crossbreeding of genetically diverse parent lines—often exhibit superior stress tolerance compared to their inbred parents [116]. The enhanced genetic diversity of hybrids confers adaptability to varying environmental conditions, including heat and waterlogging [117]. Maize and rice hybrids developed through this approach have shown improved yields, vigorous root growth, and enhanced aerenchyma development under stress [118,119].

Successful breeding programs have led to the advancement of crops with improved tolerance to heat and waterlogging. The Drought-Tolerant Maize for Africa (DTMA) initiative produced hybrids with greater root biomass and improved osmotic adjustment, significantly enhancing drought resilience and yields in Sub-Saharan Africa [120]. Similarly, in rice, the development of Sub1 varieties carrying the Sub1 gene represents a milestone in breeding for submergence tolerance. These varieties can withstand complete submergence for up to two weeks, making them particularly valuable in flood-prone regions [121]. Furthermore, In carrot (*Daucus carota*), selection for deeper root systems improved both drought tolerance and nutrient uptake under water limitation [122]. Moreover, in pea (Pisum sativum), breeding efforts have produced varieties with improved root aeration and regeneration after flooding, ensuring stable production in rainfall-intensive regions [123].

Altogether, these examples demonstrate that conventional breeding, through trait selection and exploitation of genetic diversity, continues to make significant contributions to enhancing crop resilience against heat and waterlogging stress. Such efforts play a vital role in sustaining food security in regions most vulnerable to climate variability.

### 4.2. Genetic Engineering Approaches

Genetic engineering provides powerful tools for enhancing tolerance to heat and waterlogging stress by directly manipulating genes that regulate stress-response pathways. The advent of genome-editing platforms such as CRISPR-Cas9 has revolutionized plant breeding by enabling precise modification of loci associated with resilience to heat, drought, and flooding [124].

For heat stress, one of the most common strategies involves the modification of heat shock proteins (HSPs), which protect cellular structures under elevated temperatures. Overexpression of *HSP101* in *Arabidopsis thaliana* improved protein stability and survival under heat stress [125]. Similarly, introduction of the tomato *LeHSP21* gene into tobacco (*Nicotiana tabacum*) enhanced thermotolerance by boosting antioxidant activity and proline accumulation [126]. Heat shock transcription factors (HSFs) also play a central role in regulating HSP networks. For example, *ZmHsfA1* of maize and *OsHsfA2c* of rice enhanced thermotolerance in transgenic *Arabidopsis* and rice by activating downstream HSPs and protective metabolic pathways [127,128].

Overexpression of *GmHSFA2* from soybean to Arabidopsis improved high-temperature tolerance by activating HSPs such as *HSP20* and protective pathways. Other stress-related genes, such as mitogen-activated protein kinases (MAPKs) and DREB transcription factors, have also been employed to improve heat tolerance. Overexpression of *SlMAPK3* in tomato and *OsMAPK3* in rice conferred heat tolerance by activating antioxidant defenses, improving photosynthetic stability, and upregulating HSP expression [129,130,131]. Similarly, *DREB1A* and *DREB2A* introduced from Arabidopsis into tomato and maize, enhanced stress-responsive gene expression and improved plant growth under elevated temperatures, respectively [132,133].

On the other hand, waterlogging stress causes significant damage to plants by depriving roots of oxygen in flooded soils, often leading to root death and inhibited plant growth. To address this, genetic engineering has targeted genes involved in submergence tolerance and ethylene signaling. The Sub1A gene, which regulates submergence tolerance in rice, has been successfully incorporated into other rice varieties, allowing them to survive flooding conditions for up to two weeks without significant yield loss [134]. Ethylene-responsive transcription factors [SNORKEL1 and SNORKEL2 (SK1/SK2)] in rice have been shown to mediate internode elongation under flooding conditions, thereby enhancing the survival of deep water rice during prolonged submergence [135]. In banana, overexpression of MaERFII3 in Arabidopsis enhanced tolerance by stimulating root growth under hypoxia [136]. Similarly, the bHLH transcription factor (CabHLH18) from waterlogging-tolerant pepper improved plant vigor, osmolyte accumulation, and antioxidant activity while reducing oxidative stress [137]. Overexpression of AvERF75 in kiwifruit (*Actinidia valvata*) further highlighted the role of ERF transcription factors by improving adaptive root and shoot responses through interaction with AvLOB41 [138]. In maize, targeted expression of genes promoting aerenchyma formation has been shown to enhance root oxygen availability and improve yield stability under waterlogged conditions [139].

Altogether, these examples demonstrate how genetic engineering complements traditional breeding by introducing precise modifications that enhance stress tolerance. The integration of HSPs, HSFs, MAPKs, DREBs, and ERFs into crop improvement strategies highlights the potential of molecular tools to develop resilient cultivars capable of maintaining yield under heat and waterlogging stress.

### 4.3. Transcriptomic Approaches to Identify Key Stress-Responsive Genes

Transcriptomic (RNA-Sequencing) analysis has become a vital tool for understanding plant responses to environmental stresses such as heat and waterlogging. This method provides a comprehensive overview of genome-wide gene expression under specific stress conditions, allowing for the identification of key regulatory genes (common and differentially expressed) and pathways involved in stress adaptation. Unlike traditional targeted gene studies, transcriptomic approach captures the dynamic expression patterns of thousands of genes simultaneously, offering insights into complex regulatory networks that govern plant stress responses [140]. The ability to compare gene expression between tolerant and sensitive genotypes makes transcriptomic analysis especially valuable.

Differential gene expression patterns can identify key genes that contribute to enhanced physiological performance under adverse conditions. By integrating transcriptomic data with physiological and biochemical analyses, researchers can link molecular changes to functional outcomes such as improved photosynthetic efficiency, osmotic homeostasis, ROS detoxification, and root survival under hypoxic conditions. Table 6 provides an overview of the key genes identified through transcriptomic analysis associated with heat and waterlogging stress tolerance in different crops.

In response to heat stress, a comparative transcriptome analysis of heat-resistant and heat-sensitive maize revealed upregulation of heat shock factors HSFs and DREBs in the tolerant genotype, leading to increased expression of heat shock proteins and antioxidant enzymes, which enhanced photosynthetic efficiency and ROS detoxification, highlighting key genes for genetic improvement [141]. Similarly, in grapevine, variations in the transcription factor *HSFA2* were linked to enhanced thermotolerance, with tolerant genotypes showing higher *HSFA2* expression under heat stress, activating heat shock proteins and protective pathways, making *HSFA2* a key target for improving heat resilience [142]. In tea, transcriptomic analysis under heat stress identified upregulation of *CsHSFA2*, *CsHSP70*, and *CsSOD*, linked to increased accumulation of protective metabolites like flavonoids and proline, suggesting potential genes for heat tolerance improvement [143]. Similarly, In eggplant, the upregulation of *SmHSP70* and *SmHSP90* in heat-tolerant cultivars, along with the enhanced expression of *IaHSP70*, *IaHSP90*, and *IaHSFA2* in water spinach, were crucial for maintaining protein stability and thermotolerance, highlighting their role in heat resilience [144,145].

Similarly, in response to waterlogging stress, transcriptomic studies have also identified key regulatory genes that contribute to stress adaptation. In barley (*Hordeum vulgare*), tolerant genotypes exhibited upregulation of genes associated with ethylene signaling, anaerobic metabolism, and ROS detoxification, such as *HvERF1*, *HvADH1*, and *HvSOD1* [146]. A comparative transcriptome analysis in chrysanthemum revealed that tolerant cultivars exhibited increased expression of genes involved in ethylene signaling, antioxidant defense (*CmSOD*, *CmCAT*), and anaerobic metabolism (*CmADH1*), while sensitive cultivars showed weaker responses, highlighting these genes as potential targets for enhancing waterlogging tolerance [147]. In melon (*Cucumis melo*), waterlogging induced upregulation of genes involved in ethylene and auxin signaling, anaerobic respiration (CmADH, CmPDC), and antioxidant defense (*CmSOD*, *CmCAT*), offering key targets for tolerance enhancement [148]. A comparative analysis of apple rootstocks also identified genes associated with ethylene and auxin signaling, carbohydrate metabolism, and ROS scavenging (*MdADH*, *MdPDC*, *MdSOD*), revealing regulatory pathways that support waterlogging tolerance and suggesting potential candidates for breeding and genetic improvement [149].

In brief, transcriptomic approaches provide invaluable insights into the molecular mechanisms that enable plants to adapt to heat and waterlogging stresses. These analyses not only enhance our understanding of gene expression patterns and regulatory pathways but also lay the foundation for developing stress-resilient crops. By integrating transcriptomic data with physiological and biochemical insights, researchers can identify hub genes and regulatory modules for targeted improvement in breeding programs, ultimately contributing to the development of more resilient agricultural systems capable of withstanding the challenges posed by climate change for heat and waterlogging stress tolerance in crops.

**Table 6:** Key genes identified through transcriptomic approaches for heat and waterlogging stress tolerance in crops.

Gene Name	Stress Type	Crop Name	Gene Function	Reference
*LlHsfA3A*, *LlHsfA3B*	Heat stress	*Lilium longiflorum*	*LlHsfA3A*: Enhances thermotolerance. *LlHsfA3B*: Improves acquired thermotolerance. Both affect proline metabolism.	[150]
*VvbZIP60*, *VvbZIP60s*	Heat stress	*Vitis vinifera*	*VvbZIP60*: Regulates heat tolerance via UPR. *VvbZIP60s*: Unconventional form that interacts with *VvHSP83*.	[151]
*CsHSFA2*, *CsHSP70*, *CsSOD1*, *CsSOD2*	Heat stress	*Camellia sinensis*	*CsHSFA2*: Responds to heat stress. CsHSP70: Stabilizes proteins. CsSOD1/CsSOD2: Detoxifies ROS.	[143]
*SmHSP70*, *SmHSP90*	Heat stress	*Solanum melongena*	*SmHSP70/90*: Stabilizes proteins under heat stress.	[144]
*IaHSP70*, *IaHSP90*, *IaHSFA2*	Heat stress	*Ipomoea aquatica*	*IaHSP70/90*: Stabilize proteins. *IaHSFA2*: Regulates heat stress.	[145]
*SlyHSFs-1*, *SlyHSFs-2*, *SlyHSFs-8*, *SlyHSFs-5*,	Heat stress	*Solanum lycopersicum*	*SlyHSFs*: Regulates heat tolerance pathways.	[152]
*MdHSFA2*, *MdGols4/6*	Heat stress	*Malus domestica*	*MdHSFA2*: Regulates heat tolerance by activating heat stress-responsive genes. *MdGolS4/6*: Involved in heat stress response.	[153]
*HvERF1*, *HvADH1*, *HvSOD1*	Waterlogging stress	*Hordeum vulgare*	*HvERF1*: Involved in ethylene signaling. *HvADH1*: Supports anaerobic metabolism. *HvSOD1*: Detoxifies ROS under waterlogging stress.	[146]
*CmSOD*, *CmCAT*, *CmADH1*	Waterlogging stress	*Chrysanthemum morifolium*	*CmSOD*: Detoxifies ROS. *CmCAT*: Breaks down hydrogen peroxide. *CmADH1*: Supports anaerobic metabolism.	[147]
*CmADH*, *CmPDC*, *CmSOD*, *CmCAT*	Waterlogging stress	*Cucumis melo*	*CmADH*: Alcohol dehydrogenase for anaerobic respiration. *CmPDC*: Pyruvate decarboxylase. CmSOD: Detoxifies ROS. *CmCAT:* Reduces hydrogen peroxide.	[148]
*MdADH*, *MdPDC*, *MdSOD*	Waterlogging stress	*Malus domestica*	MdADH: Alcohol dehydrogenase for anaerobic metabolism. *MdPDC*: Pyruvate decarboxylase. MdSOD: Detoxifies ROS under waterlogging stress.	[149]
*CabHLH18*	Waterlogging stress	*Capsicum annuum*	*CabHLH18*: A bHLH transcription factor that improves growth, root length, and stress-related metabolites under waterlogging stress.	[137]
*OsSub1A*, *OsERF71*	Waterlogging stress	*Oryza sativa*	OsSub1A: Regulates ethylene response and anaerobic metabolism. *OsERF71*: Involved in submergence tolerance	[154]

### 4.4. Metabolic Insights into Stress-Responsive Pathways

Metabolomics is a powerful and comprehensive approach used to study the small molecules, or metabolites, present in plants, which are crucial for understanding how plants respond to environmental stresses [155]. Unlike transcriptomics, which focuses on gene expression, metabolomics provides a direct measure of the biochemical changes occurring within the plant system. By analyzing metabolites, researchers can uncover the dynamic adjustments plants make to maintain homeostasis and survival under stress conditions. These adjustments often involve the synthesis and accumulation of osmolytes, secondary metabolites, sugars, amino acids, and other small molecules that play critical roles in stress tolerance [156,157]. Metabolomics also enables the identification of antioxidants and hormones that facilitate the mitigation of oxidative stress and the activation of signaling pathways necessary for stress adaptation [155,158]. Meanwhile, the key metabolites and metabolic pathways identified through metabolomic approaches in response to heat and waterlogging stress in various crops are summarized in Table 7.

Metabolomics analysis of plants under heat stress revealed significant alterations in carbohydrate metabolism, specifically in the levels of sucrose, glucose, and starch, which contribute to thermotolerance by maintaining energy homeostasis and osmotic regulation, while protecting cellular structures from heat-induced damage [159]. In maize (*Zea mays*), proline was identified as a critical metabolite for heat stress tolerance, where its accumulation helped maintain osmotic balance, stabilize proteins, and prevent oxidative damage under high-temperature conditions [160]. Comparative transcriptomic–metabolomic studies in lettuce (*Lactuca sativa*) under heat stress showed increased proline, glucose, fructose, and flavonoids, which supported osmotic adjustment, oxidative stress protection, and cellular stabilization, thereby enhancing thermal resistance and identifying potential breeding targets [161]. Similarly, in Arabidopsis, metabolomic profiling during germination and seedling growth under heat stress revealed changes in lipid composition, together with elevated proline, sucrose, and phospholipids, which improved membrane stability, energy regulation, and oxidative stress mitigation [162]. In soybean (*Glycine max*), exposure to combined drought and heat stress altered sugar and nitrogen metabolism, with higher levels of glucose, fructose, sucrose, proline, and glutamine. These metabolites contributed to osmotic regulation, sustained energy production, and protein stability, reflecting key metabolic adjustments for tolerance to multiple stresses [163].

Similarly, under waterlogging stress, metabolomic profiling has revealed major adjustments that support plant survival under hypoxic or anoxic conditions. In sesame (*Sesamum indicum*), metabolomic analysis under waterlogging stress revealed an increased levels of antioxidant metabolites such as ascorbate peroxidase (APX) and glutathione (GSH), together with changes in glutathione, glyoxylate, and dicarboxylate metabolism, were linked to oxidative stress mitigation and enhanced tolerance [164]. In barley (*Hordeum vulgare*), tolerant genotypes displayed higher sugar content, reduced lactate accumulation, and stronger ethanol fermentation activity, sustaining energy supply and alleviating oxidative damage compared with sensitive lines [165]. In quinoa (*Chenopodium quinoa*) seedlings under waterlogging stress, metabolomic analysis identified increased sucrose, glucose, and trehalose to sustain energy production, while GABA and phenolics enhanced antioxidant defense and root survival under hypoxia condition [166]. In *Ophiopogon japonicas*, combined transcriptomic and metabolomic analyses revealed elevated flavonoids (apiin and pelargonin) and regulation by C_2_H_2_ zinc finger and AP2/ERF transcription factors, highlighting genetic–metabolic interactions in waterlogging adaptation [167]. Metabolite studies in *Brassica napus* showed that the tolerant genotype G230 accumulated higher levels of naringenin, epiafzelechin, and pyridoxal phosphate compared with the sensitive genotype G218, linking these metabolites to waterlogging resistance [168].

In short, metabolomics studies provide critical insights into the biochemical reprogramming that drives plant adaptation to heat and waterlogging stress. Key metabolites, including osmolytes, antioxidants, and secondary compounds, regulate osmotic balance, stabilize proteins, and mitigate oxidative damage, thereby sustaining cellular function under stress. When integrated with transcriptomic and physiological data, metabolomics not only identifies reliable biomarkers of tolerance but also guides breeding and genetic engineering strategies. These advances establish metabolomics as a powerful approach for both crop improvement and a deeper understanding of plant stress biology.

**Table 7:** Key metabolites and pathways identified through metabolomics approaches in response to heat and waterlogging stress.

Metabolite Name	Stress Type	Crop Name	Metabolic Pathway/Function	Reference
Proline	Heat stress	*Zea mays*	Maintains cellular hydration and acts as an antioxidant, protecting against heat stress	[160]
Phenylpropanoid	Heat stress	*Cucumis sativus*	Phenylpropanoids, including flavonoids, accumulate under heat stress, contributing to stress tolerance.	[169]
Glutathione, Amino Acids, Organic Acids, Flavonoids, Sugars	Heat stress	*Capsicum annuum*	Accumulation of glutathione, amino acids, organic acids, flavonoids, and sugars enhances heat stress tolerance by regulating osmotic balance and providing protective effects.	[170]
Cytokinins, Auxins, Gibberellins, Abscisic Acid, Salicylic Acid Ethylene	Heat stress	*Eriobotrya japonica*	Cytokinins, auxins, gibberellins, abscisic acid, salicylic acid and ethylene regulate heat stress by modulating stress tolerance pathways.	[171]
Glutathione, Nucleoredoxin, GABA pathway metabolites	Heat stress	*Mangifera indica*	Glutathione detoxifies ROS, nucleoredoxin and GABA-shunt metabolites help regulate oxidative stress.	[172]
Malondialdehyde (MDA), Superoxide Dismutase (SOD), Guaiacol Peroxidase (POD), Catalase (CAT)	Waterlogging stress	*Citrus sinensis*	MDA is a marker for oxidative stress; SOD, POD, and CAT detoxify ROS under waterlogging stress	[173]
Peroxidase (PERs), Cinnamoyl-CoA Reductases (CCRs)	Waterlogging stress	*Hordeum vulgare*	Peroxidases (PERs) and Cinnamoyl-CoA reductases (CCRs) enhance cell wall biogenesis and peroxidase activity in the phenylpropanoid pathway thus improving waterlogging tolerance.	[165]
GABA, Sucrose, Glucose, Trehalose	Waterlogging stress	*Chenopodium quinoa*	GABA regulates phenylpropanoid metabolism, enhancing flavonoid and phenolic acid biosynthesis. Sucrose, glucose, and trehalose provide energy for adventitious root production during waterlogging.	[166]
Ascorbate Peroxidase (APX), Glutathione (GSH)	Waterlogging stress	*Sesamum indicum*	APX and glutathione (GSH) are antioxidant enzymes that enhance the oxidative stress tolerance	[164]

### 4.5. Agronomic Practices for Enhancing Plant Tolerance to Heat and Waterlogging Stress

**Figure 9:**
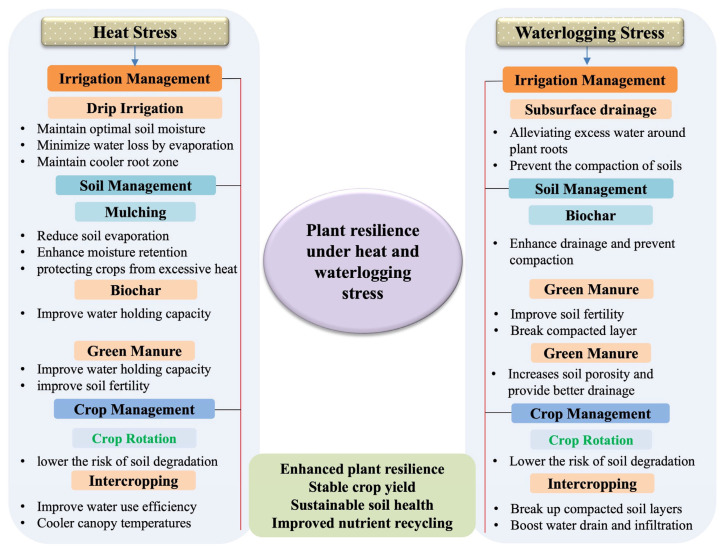
Agronomic practices for enhancing plant resilience under heat and waterlogging stress.

Agronomic practices are central to sustaining crop health under environmental stresses such as heat and waterlogging, both of which are intensifying with climate change [174]. Unlike breeding and genetic engineering, which provide long-term solutions, agronomic interventions deliver immediate and practical solutions that can be implemented on the ground by farmers to mitigate the detrimental impacts of these stresses [175]. Key approaches include water and nutrient management, soil improvement, and diversified cropping systems, all of which strengthen plant resilience [174]. As summarized in Figure 9, key agronomic practices, including irrigation management, soil management, and crop rotation/intercropping, play a crucial role in enhancing plant resilience under both heat and waterlogging stresses. This section highlights the roles of irrigation and drainage, soil conditioning, and crop rotation with intercropping in improving tolerance to heat and waterlogging.

#### 4.5.1. Irrigation and Drainage Management

Efficient water management is essential for mitigating both heat and waterlogging stress, which are among the primary environmental factors negatively impacting crop yield and overall plant health. Heat stress is a growing concern due to the increasing frequency of high-temperature events, and it leads to higher evaporative demand from crops, leading to dehydration and subsequent wilting [176]. Maintaining optimal soil moisture through precise irrigation is therefore critical. Drip irrigation, which supplies water directly to the root zone, minimizes evaporative loss, conserves water, and helps sustain cooler root temperatures, thereby lowering physiological stress during high heat [177]. In addition, surface mulching and rain shelters can complement irrigation by reducing evaporation and protecting crops from extreme sunlight, thus stabilizing the crop environment during elevated heat or drought condition [178].

Conversely, waterlogging from excessive rainfall or poor drainage creates hypoxic root conditions that disrupt respiration and nutrient uptake [179]. Subsurface drainage systems, using perforated pipes to remove excess water, are effective in maintaining oxygen availability and root function [180]. Raised-bed farming and mounded planting also mitigate waterlogging by elevating the root zone, improving aeration, and preventing soil compaction in poorly drained soils [181]. Together, these practices provide practical and immediate solutions for improving crop resilience under contrasting water-related stresses.

#### 4.5.2. Soil Management

Soil structure and composition are critical factors in determining plant resilience to environmental stresses, particularly heat and waterlogging. The physical properties of soil, including its texture, porosity, and organic matter content, directly influence water retention, drainage, and root health. When soil structure is compromised, it often leads to poor water infiltration, erosion, or compaction, which can exacerbate the impacts of heat and waterlogging stresses. Both of these environmental challenges hinder plant growth by interfering with root function, nutrient uptake, and overall metabolic processes. Therefore, enhancing soil quality through targeted management is therefore essential for stress mitigation.

One of the most effective soil management techniques for mitigating heat stress is mulching. Mulching involves covering the soil surface with organic or synthetic materials that reduce water loss by evaporation. Mulching not only limits water loss but also improves soil structure by moderating temperature and adding organic residues that contribute to long-term fertility [178]. Similarly, composting improves soil structure and water-holding capacity by increasing organic matter content, while also facilitating drainage during periods of excess rainfall [182]. Together, these practices help maintain a more stable soil environment across contrasting stress conditions.

The incorporation of green manure adds organic matter and nutrients while improving soil porosity. Their deep root systems prevent compaction, enhance infiltration, and support root growth under flooding, making soils more resilient to both drought and waterlogging [183,184]. Similarly, the application of biochar, a form of charcoal produced from organic materials through pyrolysis, significantly enhances soil structure. Biochar improves the water-holding capacity of soil by increasing its porosity, which allows for better water retention during dry periods and improves drainage during heavy rainfall, thus reducing the risk of waterlogging [185].

In summary, soil management practices—including mulching, composting, green manure, and biochar application—contribute to improved soil structure, moisture regulation, and aeration. These practices not only buffer crops against high temperatures by maintaining soil moisture and reducing heat stress, but also protect against waterlogging by facilitating drainage and minimizing compaction. Strengthening soil health through these approaches is therefore a basis of building resilient and sustainable cropping systems in the face of climate change.

#### 4.5.3. Crop Rotation and Intercropping

The practices of crop rotation and intercropping have long been recognized as effective agronomic strategies to improve soil health, optimize nutrient cycling, and mitigate environmental stresses, including heat and waterlogging [186]. Crop rotation involves cultivating different species on the same field over successive seasons, carefully choosing species with diverse rooting patterns, water needs, and stress tolerances. Rotating deep-rooted crops such as maize or sunflower with shallow-rooted crops like legumes or small grains can improve soil structure, reduce compaction, and lower the risk of soil degradation, which often worsens the effects of heat and waterlogging stress [187].

Intercropping, which involves growing two or more crops together in the same field, can further strengthen plant resilience to environmental stresses through complementary interactions. Legumes intercropped with cereals enrich soil nitrogen via biological fixation while also improving water use efficiency by moderating soil evaporation and canopy temperature [188]. The inclusion of deep-rooted or structurally distinct species improves soil aeration, water infiltration, and drainage, which is particularly beneficial under excessive rainfall and waterlogging [189,190]. Intercropping also promotes microbial diversity and activity, further enhancing nutrient availability and plant health under stress [191].

Using crop rotation and intercropping together is essential for maintaining fertile soils and resilient farming systems under extreme heat, waterlogging, and other environmental challenges [186]. These practices improve soil structure, promote efficient nutrient cycling, and sustain diverse microbial communities, thereby enhancing plant health and stress tolerance. Crop rotation, by alternating crops with contrasting rooting depths, water needs, and stress sensitivities, prevents soil degradation and mitigates adverse effects of climate variability [192]. Intercropping fosters complementary interactions among species, moderates soil and canopy microclimates, and improves water use efficiency [193]. Together, these strategies stabilize crop yields, reduce susceptibility to abiotic stress, and sustain long-term soil capacity. Integrated into broader agronomic management, they provide practical solutions to maintain crop performance under increasingly unpredictable environmental conditions. Figure 9 mainly outlines key management practices in irrigation, soil, and crop management aimed at improving crop resilience to environmental stresses. For heat stress, strategies include drip irrigation, mulching, biochar, green manure, crop rotation, and intercropping. For waterlogging stress, subsurface drainage, biochar, green manure, and crop rotation are emphasized. These practices collectively promote stable crop yields, sustainable soil health, and improved nutrient cycling.

### 4.6. Integrated Strategies for Developing Stress-Resilient Crops: Translating Comparative Insights into Targeted Interventions

#### 4.6.1. Conceptual Rationale

In the previous sections, we explored different strategies aimed at enhancing crop tolerance to the growing threats of heat and waterlogging stresses. These strategies—spanning molecular breeding, genetic engineering, and agronomic practices—offer individual solutions, each with its potential for improving plant resilience. However, the key to long-term agricultural sustainability lies in integrating these approaches to develop crops that are not only stress-tolerant but also adaptable to the increasingly unpredictable environmental challenges brought about by climate change.

Building on the comparative insights from this review, we recognize that understanding the shared biological mechanisms and distinct adaptive responses to both stresses is essential for designing effective resilience strategies. By targeting shared regulatory hubs and stacking stress-specific traits, we can cultivate crops that are genetically resilient, capable of thriving under multiple stress conditions, and optimally equipped to face the challenges of a changing climate.

Mainly, integrated strategies connect mechanistic insights from comparative analyses to practical deployment. By distinguishing stress-specific responses (e.g., HSFs or ERF-VII networks) from shared signaling hubs (e.g., Ca^2+^, ROS, MAPKs), this framework guides targeted choice of interventions based on environmental predictability and crop biology [194].

#### 4.6.2. Strategic Logic

1. Target Shared Regulatory Hubs for Broad Resilience: Central hubs like ROS-scavenging networks, calcium signaling components, and MAPK cascades operate across multiple stresses and represent high-leverage targets for broad tolerance. Enhancing these pathways strengthens basal resilience where stress occurrence is unpredictable [195]. However, the comparative framework underscores the importance of shared regulatory hubs between heat stress and waterlogging stress, including ROS scavenging, ABA signaling, and antioxidant defense systems. These mechanisms, which are activated in both stresses, present effective targets for broad resilience strategies that enable crops to withstand both environmental stressors. By focusing on these shared mechanisms, it is possible to enhance multi-stress tolerance, ensuring crops remain resilient even in unpredictable environments where both heat and waterlogging co-occur.
  A. *Molecular Breeding*
  The application of GWAS and QTL mapping allows breeders to identify genetic markers linked to shared mechanisms such as ROS detoxification and ABA-mediated regulation. By targeting these markers, breeding programs can enhance multi-stress tolerance across different environments. Tools like marker-assisted selection (MAS) and CRISPR-Cas9 technology can facilitate the integration of these traits into crops, improving their ability to respond to both stresses simultaneously. For example, optimizing ROS management pathways through genetic modification can confer resilience to both heat and waterlogging stress, benefiting crops like rice, maize, and wheat.
  B. *Agronomic Practices*
  In addition to genetic strategies, agronomic practices play a critical role in reinforcing multi-stress resilience. Practices such as optimized irrigation management, which addresses waterlogging issues, and early planting, which mitigates the effects of heat stress, can complement genetic improvements. By implementing strategic field management, such as proper irrigation timing and planting schedules, we can help crops adapt to unpredictable environmental stressors. These practices, when integrated with molecular breeding, offer a holistic approach to enhancing crop resilience in regions where both heat and waterlogging stresses are prevalent.
2. Stack Stress-Specific Traits for Predictable Co-occurrence: When heat and waterlogging reliably co-occur, combining heat-specific (HSF/HSP) and waterlogging-specific (ERF-VII, aerenchyma formation) traits maximizes adaptation tailored to local conditions [196]. In regions where heat and waterlogging stress co-occur predictably, a more effective strategy is to focus on stacking stress-specific traits that address the unique challenges posed by each stress. This approach leverages specific adaptations for each stressor to enhance crop performance under predictable stress environments. For example, combining heat shock proteins (HSPs), which improve heat tolerance, with ethylene-responsive transcription factors (ERF-VII) that promote aerenchyma formation under waterlogging stress can optimize crop resilience. By targeting these distinct mechanisms, we can tailor interventions to environments where both heat and waterlogging co-occur regularly, ensuring that crops are specifically adapted to these combined stresses.
  A. *Molecular Tools*
  Genome editing technologies such as CRISPR-Cas9 allow for precise stacking of stress-specific traits, enabling crops to adapt to co-occurring stresses with high specificity. For example, stacking HSPs for heat stress tolerance and ERF-VII transcription factors for waterlogging tolerance offers a targeted approach to improve resilience under predictable environmental conditions. This stacking strategy ensures that the plant has the necessary mechanisms to survive and thrive in both heat and waterlogging stress scenarios, which are often seen together in certain geographic regions or seasons.
  B. *Phenomics Technologies*
  Phenomics technologies, particularly high-throughput phenotyping, provide a means to screen for key traits such as root architecture, stomatal regulation, and photosynthetic efficiency under combined heat and waterlogging stresses. These phenotypic traits are crucial for ensuring optimal plant performance under both stresses, allowing breeders to select plants that exhibit the best combinations of traits for multi-stress resilience. Phenomics thus accelerates the process of identifying stress-resilient plants, allowing for faster development of crops that are genetically and physiologically optimized for co-occurring stresses.
3. Implementation Flow:
  **Step 1:** Characterize environmental stress patterns (heat only, waterlogging only, combined): Conduct a thorough assessment of the frequency, intensity, and timing of heat and waterlogging events in the target environment. Use historical climate data, predictive modeling, and in-field measurements to determine whether stresses occur independently or concurrently. For instance, in regions with seasonal monsoons followed by heat waves, crops may require stacked adaptations, while areas with sporadic heat events may benefit more from shared hub optimization. This step ensures that subsequent interventions are environmentally contextualized rather than generic [197].
  **Step 2:** Map key stress-specific and shared mechanisms: Identify the molecular, hormonal, physiological, and morphological pathways underlying tolerance to heat, waterlogging, and shared stress responses. Heat-specific mechanisms include HSF-HSP networks, ABA-mediated stomatal regulation, and thermos-tolerance-associated metabolites [198,199]. Waterlogging-specific mechanisms include ERF-VII transcription factors, aerenchyma and adventitious root formation, and anaerobic metabolism [200]. Shared hubs involve ROS scavenging, Ca^2+^-MAPK signaling, and antioxidant enzyme activation [195,198,199,200]. Mapping these pathways allows prioritization of targets for breeding, genome editing, or agronomic interventions.
  **Step 3:** Prioritize interventions targeting shared hubs or stress-specific stacks. For unpredictable or variable stress environments, prioritize interventions that enhance shared regulatory hubs to provide moderate tolerance to multiple stresses simultaneously [194,198]. For predictable dual-stress environments, implement trait stacking: combine stress-specific adaptations (e.g., HSP induction + ERF-VII-mediated root adaptations) to maximize resilience [197,201,202].
  Consider potential synergistic or antagonistic interactions between traits—for example, overexpressing HSPs may influence ROS dynamics, which also impact waterlogging tolerance—requiring integrated evaluation.
  **Step 4:** Select deployment pathways (breeding, editing, and agronomy) based on crop species and resource context. Decide whether interventions are best applied through molecular breeding, transgenic/genome-editing approaches, or agronomic management. Molecular breeding is suited for crops with extensive genomic resources and for stacking multiple loci. Transgenic or genome-editing approaches are optimal for precise modifications of key hubs, especially where trait stacking is required. Agronomic interventions—such as optimized irrigation, raised beds, or priming treatments—can complement genetic improvements and provide immediate stress mitigation [195,203]. The chosen deployment pathway should also consider economic feasibility, farmer adoption potential, and regulatory constraints.

#### 4.6.3. Decision Matrix for Resilience Strategies

Here are some proposed resilient strategies for plants based on responsive mechanisms towards heat and waterlogging stress (Table 8).

## 5. Conclusions

An effective level of tolerance to heat and waterlogging stress in plants requires a coordinated optimization of shared regulatory hubs—notably ROS scavenging, calcium signaling, and MAPK cascades—alongside stress-specific core solutions, including chaperone networks for heat and anaerobic metabolic adaptations for waterlogging. Our comparative analysis demonstrates that these mechanisms operate hierarchically across molecular, hormonal, physiological, and morphological levels, and that the interplay between stress-specific and shared pathways dictates adaptive success. By integrating molecular insights with physiological outcomes, this review highlights that broad resilience cannot be achieved by targeting a single stress pathway; rather, a holistic strategy that balances convergent and unique mechanisms is essential for sustained plant performance under dynamic environmental conditions.

## 6. Future Directions

**1. Molecular Network Elucidation under Combined Stress:** While ROS, calcium, and MAPK hubs are central to shared stress responses, their dynamic cross-talk under simultaneous heat and waterlogging remains poorly understood. Future studies should integrate multi-omics and time-resolved phenotyping to map these interactions and identify bottlenecks for resilience enhancement.

**2. Targeted Trait Stacking for Predictable Stress Combinations:** Most current breeding and engineering efforts focus on single-stress adaptation. There is a need for systematic stacking of heat- and waterlogging-specific traits (e.g., HSP/HSF and ERF-VII networks) with shared hub optimization, guided by environmental modeling of stress co-occurrence.

**3. Translational Approaches Linking Mechanisms to Field Performance:** Linking molecular and physiological insights to field-level outcomes remains a bottleneck. Precision agriculture, high-throughput phenotyping, and predictive modeling should be used to translate mechanistic findings into crop management strategies and cultivar deployment.

**4. Exploration of Understudied Hormonal Crosstalk and Epigenetic Regulation:** Hormonal interactions, particularly ABA–ethylene–GA networks, and epigenetic modifications under dual stress conditions are emerging targets for broad resilience but require further experimental validation.

**5. Development of Integrated Decision Frameworks for Stress Management:** Future work should expand upon the decision matrix proposed here, incorporating real-world data on stress predictability, environmental heterogeneity, and crop-specific responses. Such frameworks will guide both breeding and agronomic interventions in a predictive and adaptive manner.

By addressing these areas, future research can translate the mechanistic insights revealed through comparative analyses into practical strategies for developing crop varieties with durable tolerance to heat, waterlogging, and their co-occurrence, ultimately supporting global food security under increasingly variable climates.

## Figures and Tables

**Table 1:** Comparative disruption of core biological processes under heat and waterlogging stress.

Mechanism/Process	Heat Stress	Waterlogging Stress	Comparative Insight/Biological Significance
Oxidative Stress	Increased ROS production in chloroplasts and mitochondria, leading to lipid peroxidation and chlorophyll degradation.	ROS production in roots due to hypoxia, causing mitochondrial dysfunction and anaerobic metabolism.	Both stresses induce ROS, but the organ-specific ROS sites differ: chloroplasts/mitochondria vs. roots. ROS management is critical under both stresses.
Respiration	Increased initially, but inhibited at high temperatures, leading to energy loss.	Reduced due to oxygen deprivation, inhibiting root growth.	Both stresses impair energy production, but mechanisms differ: thermal inhibition vs. hypoxic limitation.
Photosynthesis	Reduced CO_2_ assimilation due to stomatal closure and damaged photosynthetic enzymes.	Reduced photosynthesis due to stomatal closure, mesophyll conductance impairment, and chlorophyll degradation.	Stomatal closure is common, but mesophyll effects and enzyme stability differ; both result in lowered carbon assimilation.
Hormonal Regulation	ABA regulates stomatal closure; HSFs activate heat shock proteins for protection.	ABA regulates stomatal closure; ethylene regulates aerenchyma formation in roots.	ABA-mediated stomatal regulation is shared; stress-specific hormonal pathways (HSFs vs. ethylene) enable adaptation.
Energy Metabolism	Increased respiration to meet energy demands, but inhibited at high temperatures.	Oxygen deprivation restricts ATP production, inhibiting root growth and nutrient uptake.	Both stresses limit energy availability; heat increases demand, waterlogging limits production.
Stress-Specific Adaptations	Heat shock proteins (HSPs) protect cellular components; HSFs regulate their expression.	Ethylene-responsive transcription factors (ERF-VII) mediate root adaptations and aerenchyma formation.	Unique adaptive strategies reflect stress type: protein protection under heat vs. structural adaptation under waterlogging.

**Table 2:** Comparative insight of stomatal regulation under heat and waterlogging stress.

Feature	Heat Stress	Waterlogging Stress
Primary trigger	Elevated temperature and vapor pressure deficit (VPD)	Root hypoxia and impaired water uptake
Key hormone involved	Abscisic acid (ABA)	Abscisic acid (ABA) and ethylene
Mechanism of closure	ABA-mediated ROS and Ca^2+^ signaling in guard cells	ABA accumulation due to root-zone hypoxia and ethylene
Photosynthetic impact	Reduced CO_2_ assimilation; increased risk of photoinhibition	Decreased gas exchange; chlorophyll degradation and leaf yellowing
Adaptive benefit	Limits transpiration water loss under high heat load	Conserves water despite saturated soil conditions
Long-term limitation	Reduced carbon fixation; potential overheating and growth suppression	Early senescence; reduced productivity due to prolonged gas exchange restriction

**Table 3:** Comparative features of schizogenous and lysigenous aerenchyma formation in plants.

Feature	Schizogenous Aerenchyma	Lysigenous Aerenchyma
Formation mechanism	Separation of pre-existing cells	Programmed cell death (PCD) and cell lysis
Energy cost	Lower	Moderate, due to enzymatic degradation
Species distribution	Less common; observed in some wetland species	Common in cereals like rice, maize, wheat, barley
Trigger	Sometimes constitutive	Induced by waterlogging, ethylene, ROS
Main regulator	Developmental control	Ethylene, ROS, NO, Ca^2+^ signaling
Adaptation benefit	Facilitates internal air movement	Reduces root respiration demand, enhances oxygen diffusion

**Table 4:** Antioxidant defense systems in plants under waterlogging and heat stress.

Antioxidant Mechanism	Key Components	Functions	References
Enzymatic Antioxidants	Superoxide dismutase (SOD), Ascorbate peroxidase (APX), Catalase (CAT), Glutathione reductase (GR), etc.	Catalyze reactions to neutralize ROS (e.g., superoxide, hydrogen peroxide)	[20,66]
Non-Enzymatic Antioxidants	Ascorbate (AsA), Glutathione (GSH), Carotenoids, Tocopherols	Scavenge ROS directly, protecting cellular components from damage	[21]
ROS Generation and Scavenging	Hydrogen Peroxide (H_2_O_2_), Superoxide (O_2_^−^), Hydroxyl radical (-OH)	Generated under stress; involved in signaling and oxidative damage	[67]
ROS Signaling Pathways	MAPK, Ca^2+^ signaling, H_2_O_2_ signaling	Regulate stress responses and acclimation processes	[70]
Plant Hormones and ROS Interaction	Abscisic Acid (ABA), Ethylene (ET)	Enhance stress tolerance via cross-talk with ROS	[71]

**Table 5:** Hormonal crosstalk and regulatory networks under heat and waterlogging stress.

Hormone	Primary Role	Stress Condition	Interaction with Other Hormones	Key Function
Abscisic Acid (ABA)	Stress response regulator	Heat and Drought	Work with ethylene	Promotes stomatal closure, Regulate root development, osmotic adjustments
Ethylene	Stress adaptation in low oxygen	Waterlogging (hypoxia)	Works with ABA	Forms aerenchyma tissue, aids in oxygen exchange, regulates stress tolerance
Gibberellins (GA)	Growth regulation	All stresses	Reduced activity during stress	Limits growth to conserve resources
Auxins	Root architecture regulation	Waterlogging	Interacts with cytokinins	Promotes root elongation and lateral root growth
Cytokinin	Cell division, shoot growth	All stresses	Works with auxins	Promotes cellular repair, stress tolerance mechanisms

**Table 8:** Decision matrix criteria for adopting the strategies to enhance crop resilience towards heat and waterlogging stresses.

Criteria	Broad Resilience (Shared Regulatory Hubs)	Stress-Specific Adaptations (Stacking Traits)
Predictability of Stress Co-occurrence	Unpredictable (Stresses occur at different times in varying region)	Predictable (Stresses co-occur in specific regions/seasons)
Target Mechanisms	ROS Scavenging, ABA Signaling, Antioxidant Systems: These shared mechanisms help manage oxidative stress and osmotic balance across different stress types, offering broad resilience to unpredictable stress environments.	HSPs for Heat Stress, ERF-VII for Waterlogging: These stress-specific adaptations target mechanisms critical to each stress, ensuring that plants can adapt effectively when both stresses co-occur in specific regions or seasons.
Application Strategy	Multi-Stress Resilience Across Various Regions: Targeting shared mechanisms like ROS scavenging and ABA signaling provides resilience against multiple stressors in varying environments	Tailored Solutions for Specific Stress Co-occurrence: Focusing on stacking traits (e.g., HSPs and ERF-VII) provides a customized solution for areas where heat and waterlogging predictably occur together.
Best Approach	Target shared mechanisms for broad resilience: By improving the plant’s ability to handle multiple stressors through shared regulatory hubs, crops become more resilient across a wide range of environmental conditions.	Stack stress-specific traits for local adaptation: By combining heat tolerance traits (HSPs) with waterlogging adaptations (ERF-VII), crops are optimized for the specific challenges of environments where these two stresses co-occur regularly.

## Data Availability

No new data were created or analyzed in this study.
